# Filarial Orchitis due to *Wuchereria bancrofti* Masquerading as Testicular Neoplasm

**DOI:** 10.4269/ajtmh.16-0038

**Published:** 2016-09-07

**Authors:** Shashikant C. U. Patne, Mayurakshi Das, Richa Katiyar

**Affiliations:** ^1^ Department of Pathology, Institute of Medical Sciences, Banaras Hindu University, Varanasi, Uttar Pradesh, India

**Figure 1.**
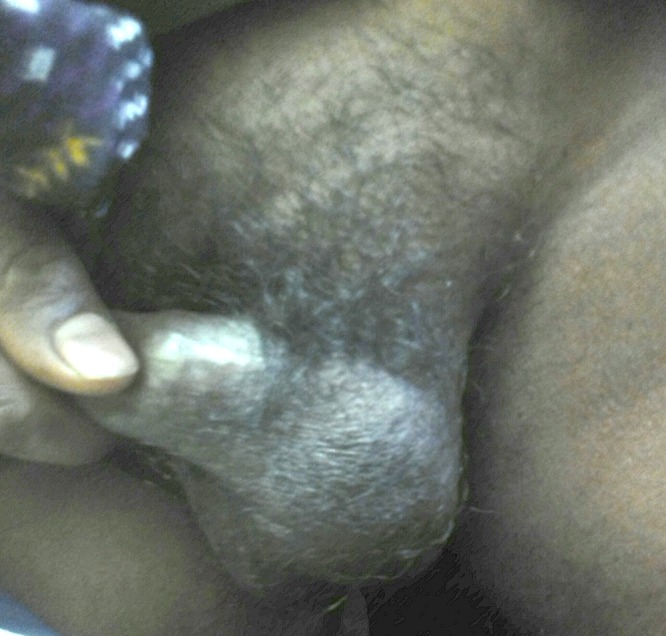
Clinical photograph of the patient showing mild enlargement of the left testis.

**Figure 2.**
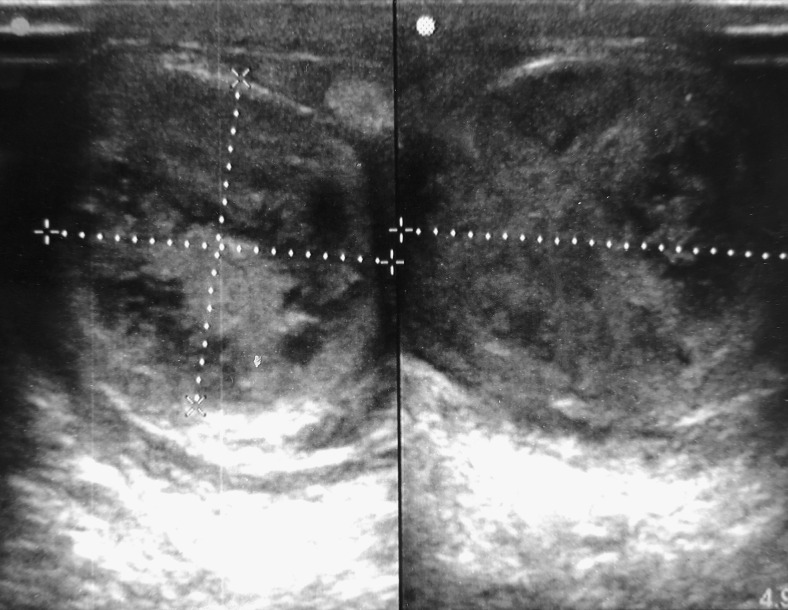
Ultrasound image showing intratesticular hypoechoic areas with mottled hyperechogenicity.

**Figure 3.**
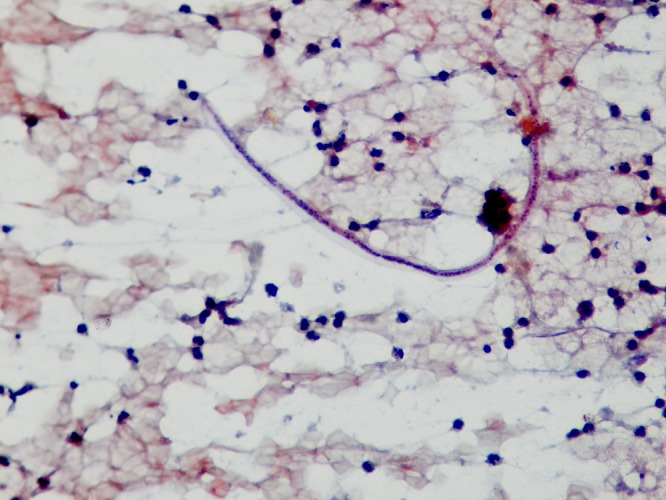
Sheathed microfilaria consistent with morphology of *Wuchereria bancrofti* admixed with neutrophils (Papanicolaou stain, ×400).

A 37-year-old man from eastern Uttar Pradesh, India, presented with 20 days of a painless swelling of the left testis. There was no history of trauma, fever, infertility, lymphadenitis, lymphangitis, or previous history of testicular tumor. On examination, the left testis measured 5×4 cm, and was found to be slightly enlarged, firm, and nontender on palpation (Figure 1

). Palpation of the right testis and bilateral spermatic cords were unremarkable. There was no inguinal lymphadenopathy. His complete blood counts were within the normal limits; differential count showed 9% eosinophils (absolute eosinophil count = 870/μL). The patient was amicrofilaremic on peripheral blood smears examined at 10 am. Results for serum alpha-fetoprotein, β-human chorionic gonadotropin, and carcinoembryonic antigen were within normal limits. Ultrasonography of the left testis showed increased vascularity and presence of hypoechoic areas containing mottled hyperechogenicity (Figure 2

). The right testis, bilateral epididymis, and bilateral spermatic cords were of normal size, shape, and echotexture. Considering clinical presentation, age, and radiological findings of the patient, a provisional diagnosis of the left testicular neoplasm (teratoma) was made, and he was advised to undergo orchiectomy. However, owing to the patient's refusal of the orchiectomy, trans-scrotal fine-needle aspiration cytology (FNAC) was done as a second line of investigation. Papanicolaou- and Giemsa-stained smears revealed a dense inflammatory infiltrate comprised of neutrophils, histiocytes, and occasional multinucleate giant cells along with many sheathed microfilarial larvae without nuclei at the tail tip (Figure 3

). FNAC findings confirmed the diagnosis of filariasis due to *Wuchereria bancrofti*. There was no evidence of malignant cells or fungal elements in the smears examined. The final diagnosis was bancroftian filarial orchitis. The patient was prescribed a 14-day course of diethylcarbamazine.

In India, > 98% cases of filariasis are caused by *W. bancrofti*.^1^ Eastern Uttar Pradesh and Bihar are the most important zones in India that are endemic for lymphatic filariasis. Isolated filarial orchitis is a rare manifestation of *W. bancrofti* infection and clinically mimics testicular malignancy or testicular torsion.^1^,^2^ Genital manifestations of lymphatic filariasis are generally asymptomatic, but may present with hydrocele, lymph scrotum, genital elephantiasis, lymph varix, and chyluria.^1^ Testicular involvement in filariasis is usually secondary to epididymitis; isolated filarial orchitis is rare. Painless, solid intratesticular masses of short duration in young men should be considered malignant, with the rare exception of filarial orchitis in filariasis endemic zones. Trans-scrotal FNAC helps to establish the correct diagnosis and obviates an unnecessary orchiectomy.
